# The p53 codon 72 proline allele is endowed with enhanced cell-death inducing potential in cancer cells exposed to hypoxia

**DOI:** 10.1038/sj.bjc.6603723

**Published:** 2007-04-03

**Authors:** P Sansone, G Storci, S Pandolfi, L Montanaro, P Chieco, M Bonafé

**Affiliations:** 1 Center for Applied Biomedical Research (CRBA), St. Orsola-Malpighi University Hospital, Bologna, Italy; 2 Department of Pharmacology and Toxicology, University of Bologna, Bologna, Italy; 3 Department of Experimental Pathology, University of Bologna, Bologna, Italy

**Keywords:** p53, codon 72, polymorphism, hypoxia

## Abstract

The preferential retention of the arginine allele at the p53 codon 72 locus is commonly observed in tumours from arginine/proline heterozygotes. Considering that cancer cells are harboured in a hypoxic environment *in vivo*, we here tested the hypothesis that the p53 codon 72 proline allele confers a survival disadvantage in presence of hypoxia. Here, we show that the transient transfection of the proline allele in p53 null cancer cells exposed to low oxygen tension or to the hypoxia-mimetic drug Desferoxamine induces a higher amount of cell death than the arginine allele. Accordingly, proline allele transiently transfected cell lines express lower levels of hypoxia pro-survival genes (HIF-1*α*, carbonic anhydrase IX, vascular endothelial growth factor, heme oxygenase-I, hepatocyte growth factor receptor, vascular endothelial growth factor receptor 2), compared to those transiently transfected with the arginine allele. Further, we report that the exposure of the arginine/proline heterozygote MCF-7 breast cancer cell line to cytotoxic concentration of Desferoxamine for several weeks, gives raise to hypoxia-resistant clones, carrying the arginine, but not the proline allele. These data indicate that the p53 codon 72 proline allele is less permissive for the growth of cancer cells in a hypoxic environment, and suggest that the preferential retention of the arginine allele in the tumour tissues of arginine/proline heterozygous patients may depend upon its lowered capacity to induce cell death in a hypoxic tumour environment.

The codon 72 arginine-to-proline polymorphism at the p53 locus affects cancer development, as well as response to therapy and survival of cancer patients (Thomas *et al*, 1999; Marin *et al*, 2000; Bergamaschi *et al*, 2003; Bonafe *et al*, 2003). The N-terminal polyproline-rich domain, in which the polymorphism is harboured, interacts with numerous proteins that are active players in the process of gene transcription and cell death (Baptiste *et al*, 2002). In particular, the two p53 codon 72 alleles show a functional difference in the binding to transcription factors (e.g., TAF30II) and in the interaction with the cell death regulatory proteins MDM-2, Bcl-2, Bcl-xL and iASPP (Matlashewski *et al*, 1987; Dumont *et al*, 2003; Bonafe *et al*, 2004; Hammond and Giaccia, 2005; Bergamaschi *et al*, 2006). The arginine allele is preferentially retained (i.e., the proline allele is preferentially lost) in cancer cells and in tumour tissues of arginine/proline heterozygous cancer affected patients (Brooks *et al*, 2000; Anzola *et al*, 2003; Bonafe *et al*, 2003; Schneider-Stock *et al*, 2004). The phenomenon has been attributed to the inactivation of the pro-apoptotic TP73 gene induced by the mutated arginine, but not proline allele (Marin *et al*, 2000). In fact, several reports indicate that the arginine allele is a better inducer of apoptosis *in vitro* (Bonafe *et al*, 2002, 2004; Dumont *et al*, 2003). However, the relevance of such a functional difference between the p53 codon 72 alleles for tumour growth *in vivo* has not yet been understood. Wild-type p53 protein induces cell death in response to hypoxia (Graeber *et al*, 1996; Semenza, 2003). Clones of cells lacking active p53 have a survival advantage in a hypoxic environment (Graeber *et al*, 1996), consistently, the hypoxic environment present in the tumour mass *in vivo* is supposed to select clones carrying defective p53 (Graeber *et al*, 1996; Semenza, 2003). Interestingly, the p53-mediated suppression of hypoxia survival genes, such as carbonic anhydrase IX and vascular endothelial growth factor has been reported (Koumenis *et al*, 2001; Pal *et al*, 2001; Kaluzova *et al*, 2004). Such a repressor activity has been proposed to be more relevant than the p53-mediated induction of pro-apoptotic genes in determining cell death in presence of hypoxia (Hammond and Giaccia, 2005; Hammond *et al*, 2006). We here tested the hypothesis that the p53 codon 72 alleles differ in the capacity to induce cell death in presence of hypoxia. We found that the proline allele confers a survival disadvantage to cancer cells exposed to hypoxic environment. We speculate that such a difference will help to understand the mechanism at the basis of the preferential retention of the arginine allele in tumour tissues *in vivo.*

## MATERIALS AND METHODS

### Cell culture and reagents

All culture media (Rosewell Park Memorial Institute (RPMI)-1640, F-12K, Low glucose Dulbecco's modified Eagle's medium (DMEM)), fetal bovine serum (FBS), L-glutamine, penicillin/streptomycin were purchased from Euroclone (Milan, Italy). MCF-7 cells were grown in RPMI-1640 medium, supplemented with 10% FBS; PC-3 cells were grown in F-12K medium supplemented with 10% FBS; HEP-3B cells were grown in low glucose DMEM, supplemented with 10% FBS; MDA-MB-157 cells were grown in 50 : 50 mixture of DMEM/F-12K media, supplemented with 5% FBS. All the cultures were kept at 37°C in a 5% CO_2_-humidified atmosphere.

### Exposure to severe hypoxia and to the hypoxia mimetic desferoxamine

Severe hypoxia (<0.1% O_2_) was generated in a humidified incubator supplied with 95% N_2_/5% CO_2_ (Thermoforma, Thermo, Waltham, MA, USA). Desferoxamine (DFX) (Sigma, St. Louis, MO, USA) was used as hypoxia mimetic. MCF-7 cells were exposed to various concentrations of DFX (100–500 *μ*M), for 4 weeks. After a massive cell death, several (20) clones were isolated in 100 *μ*M administered cultures, and were genotyped for the p53 codon 72 polymorphism (Supplementary Figure 1). No clones were obtained when >100 *μ*M of DFX was used. The clone endowed with the best capacity of *in vitro* growth (clone number 7, HYPO-7) was extensively passaged and cultured for at least 1 year in absence of DFX, without appreciable changes in morphology and gene expression (Supplementary Figure 2).

### Transient transfection of the p53 codon 72 alleles encoding plasmids

Plasmids encoding the p53 codon 72 proline or arginine allele were obtained by cloning the entire p53cDNA into a pCMS-GFP expression vector (Clontech, Palo Alto, CA, USA), as described elsewhere (Bonafe *et al*, 2004). To perform transient transfection in 3 cm^2^ wells, 60% confluent cells were incubated with 800 ng of plasmid for 24 h, in the presence of the transfection reagent Lipofectamine 2000 at a ratio of 1 : 3 (Invitrogen, Carlsbad, CA, USA). Transfection efficiency was scored using fluorescent microscopy of the GFP-expressing cells.

### Stable retroviral tranduction with dominant-negative mini p53 protein

Retroviral gene transfer was performed as described previously (Per *et al*, 1993). Briefly, Phoenix cells (kindly provided by Dr K Marcu, Department of Molecular Biology, University of New York at Stony Brook, New York, USA) were grown at 85% confluence and were transfected overnight with 10 *μ*g of the retroviral pBabe-puro plasmid, either empty or encoding a dominant negative p53-miniprotein (p53D), provided by Dr M. Oren (The Weizman Institute Rehovot, Israel). P53D is a C-terminal fragment of p53 that retains the multimerisation, but not the transcriptional transactivation domain, and it forms transcriptionally inactive multimers with endogenous wild-type p53 protein, which in turn accumulate in the cells owing to the lack of MDM2-mediated degradation (Shaulian *et al*, 1992). Two days after transfection, the medium containing newly packaged retrovirus was collected and filtered through a 0.45-*μ*m pore size filter. After supplementation with 4 *μ*g ml^−1^ polybrene (Sigma), the augmented medium was applied to MCF-7 cells at 50% for 24 h. Puromycin 2 *μ*g ml^−1^ (Sigma) was used as a selection agent to isolate successfully transduced cells.

### Double-strand RNA oligonucleotide-mediated p53 gene silencing

For the oligonucleotide-mediated silencing of the p53 gene we used predesigned p53-specific short interfering double-strand RNA oligonucleotide (GCAUGAACCGGAGGCCCA sense, AUGGGCCUCCGGUUCAUG, antisense) and a non-specific short interfering RNA (siRNA) control oligonucleotide (UUCUCCGAACGUGUCACG sense, ACGUGACACGUUCGGAGA, antisense), (Qiagen, Valencia, CA, USA). Transfection procedures were performed using Lipofectamine 2000 (Invitrogen). Briefly, 60% confluent MCF-7 cells were incubated with 1 *μ*g of p53 or control siRNA for 4 h, and then incubated for additional 24 h in fresh complete RPMI-1640 medium supplemented with 10% FBS. The efficiency of gene silencing ranged between 70 and 90%.

### p53 codon 72 genotype, p53 cDNA sequencing, loss of heterozygosity at p53 locus analyses

The DNA and cDNA sequences containing the p53 codon 72 polymorphism were amplified using a standard PCR protocol with the following primers: *FW*, 5′-GAC CCAGGTCCAGATGAAGCT-3′, *RV*, 5′-ACCGTAGCTGCCCTGGTAGGT-3′. PCR conditions were as follows: initial denaturation at 94°C for 3 min, 29 cycles with denaturation at 94°C for 1 min, annealing at 63.5°C for 1 min and extension at 72°C for 1 min, followed by final extension at 72°C for 7 min. The 156 bp PCR product was digested with restriction enzyme *BstU*I (New England Biolabs, Beverly, MA, USA), which recognises a restriction site on the arginine allele, yielding a 109 bp fragment (and an undetectable band of 47 bp). The entire p53 cDNA was amplified by using the following primers, 5′-GCCATG GAGGAGCCGCAGTC-3′ and 5′-TCAGTCTGAGTCAGGCCCTT-3′. The 1189 bp fragment was sequenced in a CEQ8000 Automatic Sequencer (Beckman, Fullerton, CA, USA), using the following primers: 5′-GCCATGGAGGAGCCGCAGTC-3′; 5′-GCCCCTCCTCAGCATCTTAT-3′; 5′-TCAGTCTGAGTCAGGCCCTT-3′; 5′-GACCCAGGTCCAGATGAAGCT-3′; 5′-ACCGTAGCTGCCCTGGTAGGT-3′. The sequence analysis revealed the presence of a wild type TP53 in both MCF-7 and HYPO-7 (data not shown). Loss of heterozygosity at p53 locus was assessed by amplifying the 100–120 bp region located in Intron I of the TP53 gene, using the following primers: 5′-ACTCCAGCCTGGGCAATAAGAGCT-3′; 5′-ACAAACATCCCCTACCAAACAGC-3′. Previous reports indicate that the above region is in tight linkage with p53 codon 72 locus (Schneider-Stock *et al*, 2004). The amplified fragments were separated on an ethidium bromide-stained 2% agarose gel.

### RNA extraction and reverse transcription cDNA amplification

Total RNA was extracted from cells using TRIzol® Reagent (Invitrogen) according to the manufacturer's instructions. Reverse transcription reaction was performed in a 20 *μ*l volume with 2 *μ*g of total RNA using the M-MLV Reverse Transcriptase (Invitrogen), following the manufacturer's protocol. Oligo-(dT)_12-18_ primers (Invitrogen) were used for the first strand synthesis. The following primers were used to amplify the target genes mRNA: vascular endothelial growth factor (VEGF): FW: 5′-GAGAATTCGGCCTCCGAAACCATGAACTTTCTGCT-3′ and RV: 5′-GAGCATGCCCTCCTGCCCGGCTCACCGC-3′, annealing temperature 65°C, amplicons length: 520, 600 and 675 bp; carbonic anhydrase IX (CA-IX): FW: 5′-CAGGGACAAAGAAGGGGATGAC-3′; RV: 5′-TTGGAAGTAGCGGCTGAAGTCA-3′, annealing temperature 61°C, amplicon length 589 bp; Heme Oxigenase-I (HO-I): FW: 5′-CCCGACAGCATGCCCCAGGAT-3′; RV: 5′-GGAGTTCATGCGGGAGCGGTAGAG-3′, annealing temperature 60°C, amplicon length 549 bp; hepatocyte growth factor receptor (c-MET): FW: 5′-ACAGTGGCATGTCAACATCGCT-3′; RV: 5′-CTTAGACATCTGATGGCTCG-3′, annealing temperature 62°C, amplicon length 656 bp; vascular endothelial growth factor receptor-2 (KDR): FW: 5′-TATAGATGGTGTAACCCGGA-3′; RV: 5′-TTTGTCACTGAGACAGCTTGG-3′, annealing temperature 62 C, amplicon length 656 bp; Hypoxia Induced Factor-1alpha (HIF-1*α*): FW: 5′-GGTGAATATGTCTGGGTTGAAAC-3′, RV: 5′-TGGGACTATTAGGCTCAGGTGAA-3′, annealing temperature 56°C, amplicon length 615 bp; PUMA: 5′-CAGACTGTGAATCCTGTGCT-3′ RV: 5′-ACAGTATCTTACAGGCTGCC-3′, annealing temperature: 62°C, amplicon length: 285 bp; NOXA: FW: 5′-GTGCCCTTGGAAACGGAAGA-3′, RV: 5′-CCAGCCGCCCAGTCTAATCA-3′, annealing temperature: 64°C, amplicon length: 258 bp; BNIP3L: FW: 5′-CTCAGTCAGAAGAAGAAGTTGTAG A-3′, RV: 5′-CTCAGTCGCTTTCCAATATAGAT-3′, annealing temperature 51°C, amplicon length: 283 bp; *β*2-microglobulin (*β*2*μ*): FW:5′-ACCCCCACTGAAAAAGATGA-3′; RV: 5′-ATCTTCAAACCTCCATGATG-3′, annealing temperature 58°C, amplicon length 114 bp . All PCR protocols were performed as follows: predenaturation step at 95°C for 2 min; 28 cycles of denaturation at 95°C for 1 min, annealing at the appropriate temperature for 1 min, extension at 72°C for 1 min; final extension at 72°C for 7 min. Amplified fragments were resolved onto a 1.8% agarose gel.

### Western blot analysis

Cell lysates were prepared using coimmunoprecipitation buffer (10 mM HEPES, 142 mM KCl, 2.5 MgCl_2_, 1 mM EDTA, 1 mM DTT, 0.2% NP-40) supplemented with a protease and phosphatase inhibitors cocktail (Sigma). Total protein lysate (100 *μ*g) was loaded onto an 8% acrylamide/bis-acrylamide gel, and transferred to Hybond, nitrocellulose membrane (Amersham Biosciences, Buckingamshire, UK). Antibodies for WB analysis were directed against: p53 (DO-I Mouse Monoclonal antibody, Santa Cruz Biotechnology, Santa Cruz, CA), CA-IX (Mouse Monoclonal antibody, Clone M75, kindly provided by J Pastorek, Slovak Academy of Sciences, Bratislava, Slovak Republic), HIF-1*α* and VEGF (Rabbit Polyclonal Antibodies, Upstate, Charlottesville, VA, USA), *β*-actin (Sigma). HRP-conjugated secondary antibodies were visualised by the luminol/enhancer chemiluminescent substrate (Amersham Biosciences).

### Cell death assessment

Cell death was evaluated by propidium iodide exclusion and analysed in a FACSaria (Becton Dickinson, San Josè, CA, USA)

### Statistical analysis

Associations among variables were verified using Student's *t*-test or analysis of variance (ANOVA). Bonferroni-corrected *post hoc* test was used for pairwise comparisons following a significant *F*-test. Statistical calculations were executed with SPSS 10.1 Software Package (SPSS Inc, Chicago, IL, USA). Data in graphs are expressed as mean±s.d.

## RESULTS

### p53 promotes cell death and represses the expression of hypoxia pro-survival genes in MCF-7 cells exposed to hypoxia

**Figure 1:**
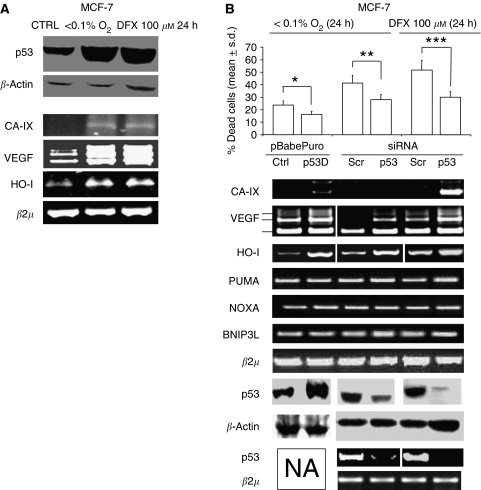
The inactivation and the downregulation of p53 enhances survival of MCF-7 cells in presence of hypoxia. (**A**) Exposure to severe hypoxia (<0.1% O_2_) or to 100 *μ*M DFX for 24 h: Western blot (WB) analysis of p53 protein, RT-PCR analysis of carbonic anhydrase IX (CA-IX), VEGF, heme oxygenase-I (HO-I) (**B**) MCF-7 cells infected with a pBabePuro retroviral vector, empty (Ctrl) or encoding a p53 dominant-negative mini-protein (p53D), or exposed to a p53-specific or control (Scr) siRNA: cell death analysis, *n*=3, ^*^P=0.008; ^**^P=0.043; ^***^P=0.015; ANOVA test, data are expressed as mean±s.d. (upper panel); RT-PCR analysis of CA-IX, VEGF, HO-I, PUMA, NOXA, BNIP3L and *β*2 *μ*m RNA level (middle panel), WB and RT-PCR analysis of p53 protein and mRNA level (lower panel).

We began this investigation by examining the role of the p53 inactivation in hypoxia-induced cell death in MCF-7. First, we generated a puromycin-resistant MCF-7 polyclonal population stably transduced with a pBabe retroviral vector, either empty or encoding a dominant-negative mini p53 protein (p53D), which hampers the activity of p53 by accumulating the protein in the cytoplasm (Shaulian *et al*, 1992). Second, we transiently transfected parental MCF-7 cells with a p53-specific short interfering RNA (p53 siRNA), which brings to a substantial reduction in the p53 mRNA level. We then exposed the cells to severe hypoxia (<0.1% O_2_) or to the hypoxia mimetic DFX, at a concentration of 100 *μ*M, and we found that both p53D-transduced cells and p53siRNA-transfected MCF-7 cells exhibited a significantly lower rate of cell death in comparison to the matched controls (Figure 1B, upper panel). The phenomenon was paralleled by a marked increase in the mRNA level of hypoxia response genes (Lee *et al*, 1997; Pal *et al*, 2001; Kaluzova *et al*, 2004) namely, VEGF, carbonic anhydrase IX (CA-IX), heme oxygenase-I (HO-I, Figure 1B, middle panel). Interestingly, no differences were observed as far as hypoxia-response/p53 regulated genes such as BNIP3L, PUMA and NOXA are concerned see Figure 1B, (middle panel). These data indicate that, in our experimental conditions, the lack of p53 activity favours hypoxia survival and prevents the downregulation of hypoxia response genes.

### The transfection of the p53 codon 72 proline allele enhances cell death in cancer cells exposed to hypoxia

**Figure 2:**
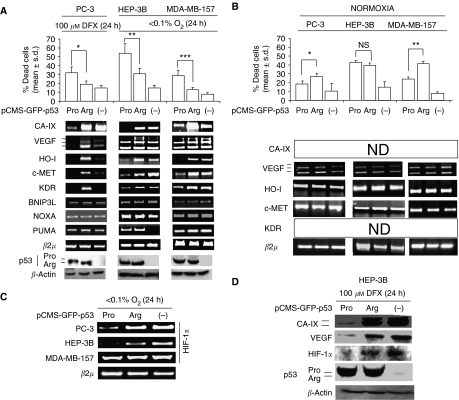
The p53 codon 72 proline allele is more cytotoxic with respect to arginine allele in presence of hypoxia. PC-3 cells exposed to 100 *μ*M DFX, HEP-3B and MDA-MB-157 exposed to <0.1% O_2_, transiently transfected with p53 codon 72 arginine- (p53Arg) or proline- (p53Pro) allele encoding, or empty (-) pCMS-GFP expression vector. (**A**) cell death analysis (*n*=3), ^*^P=0.016; ^**^P=0.006;^***^P=0.008; Bonferroni-corrected *post hoc* test, data are expressed as mean±s.d. (upper panel); RT-PCR analysis of CA-IX, VEGF, HO-I, c-MET, KDR, BNIP3L, NOXA, PUMA and *β*2 *μ*m RNA level (middle panel); Western blot analysis of p53 and *β*-actin (Note the faster migration rate of the p53Arg, with respect to the p53Pro allele, lower panel), (**B**) cell death analysis (*n*=3), ^*^P=0.049; ^**^P=0,016; NS=not significant; Bonferroni-corrected *post hoc* test; data are expressed as mean±s.d. (upper panel), RT-PCR analysis of VEGF, HO-1, c-MET, *β*2*μ* mRNA level; ND, not detectable (lower panel), (**C**) RT-PCR analysis of HIF-1*α* and *β*2 *μ*m RNA level in PC-3, MDA-157, HEP-3B cells, exposed to <0.1% O_2_ for 24 h and transfected with p53Arg or p53Pro or (-) pCMS-GFP vector. (**D**) Western blot analysis of CA-IX, VEGF, HIF-1*α*, p53, *β*-actin in HEP-3B cells, exposed to 100 *μ*M DFX for 24 h and transiently transfected with p53Arg or p53Pro or (-) pCMS-GFP vector. Transfection efficiency was evaluated by fluorescent microscopy analysis of GFP-positive cells (data not shown).

To ascertain whether the p53 codon 72 alleles show a functional difference with regards to cell survival and regulation of hypoxia response genes, p53-null cells (the breast cancer cell line MDA-MB-157, the hepatocellular carcinoma cell line HEP-3B, the prostate carcinoma cell line PC-3) were exposed to <0.1%. O_2_ and/or to 100 *μ*M DFX, and then transiently transfected with a pCMS-GFP vector, either empty (-), or encoding the p53 codon 72 arginine (p53Arg) or proline (p53Pro) allele. We found that the p53Pro allele induced a higher rate of cell death than the p53Arg allele in all the three cell lines (Figure 2A, upper panel). Moreover, though p53Arg/p53Pro transfected cells showed similar levels of p53 protein (Figure 2A, lower panel), p53Arg allele-transfected cells expressed higher levels of hypoxia survival genes, namely CA-IX, VEGF, HO-I, hepatocyte growth factor receptor (c-MET), and vascular endothelial growth factor receptor 2 (KDR), than p53Pro-transfected ones (Figure 2A, middle panel). At variance, the transfection of the p53Arg/p53Pro alleles in absence of hypoxic environment, elicited a similar degree of cell death in HEP-3B cells as well as the p53Arg allele elicited a higher degree of cell death than the p53Pro in MDA-MB-157 and PC3 cells (Figure 2B). Since the genes above are regulated by the hypoxia response transcription factor HIF-1*α* at the promoter level (Lee *et al*, 1997; Pal *et al*, 2001; Pennacchietti *et al*, 2003; Kaluzova *et al*, 2004), we then tested the hypothesis that the p53 codon 72 alleles exerts a direct regulation on HIF-1*α* gene expression. We found that, in the presence of <0.1% O_2_, p53Arg allele-transfected MDA-MB-157 and PC-3 cells, but not HEP3B, expressed a higher level of HIF-1*α* gene mRNA than p53Pro-transfected ones (Figure 2C). Nevertheless, p53Arg-transfected HEP3B cells exposed to 100 *μ*M DFX, showed increased levels of HIF-1*α*, CA-IX and VEGF protein in respect to p53Pro-transfected ones (Figure 2D). These data suggest that the p53Pro allele hampers the capacity of cancer cells in a hypoxic environment by regulating HIF-1*α* at transcriptional or post-transcriptional level, depending on the cell type examined.

### The p53Arg allele is retained in hypoxia-resistant, MCF-7 derived clones

**Figure 3:**
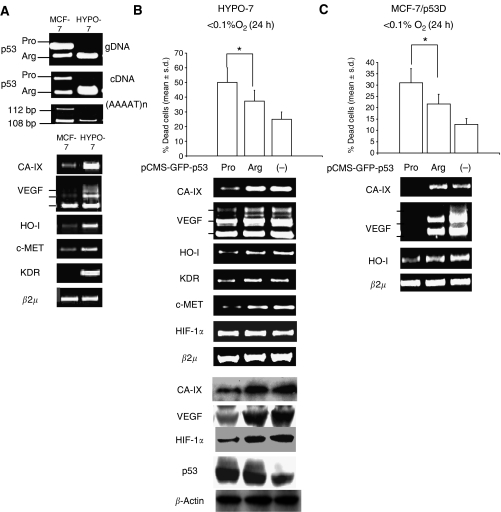
The p53Arg is retained in MCF-7-derived hypoxia-selected clones. (**A**) HYPO-7 and MCF-7 cells: PCR analysis of p53 codon 72 genotype on genomic DNA (gDNA) and cDNA, Loss of heterozygosity at the (AAAAT)_n_ pentanucleotide repeat located in the Intron I of the TP53 gene (upper); RT-PCR analysis of CA-IX, VEGF, HO-I, c-MET, KDR and *β*2 *μ*m RNA level (lower). (**B**) HYPO-7 cells exposed to <0.1% O_2_ for 24 h and transiently transfected with p53Arg, p53Pro (-) pCMS-GFP vector: Cell death analysis (*n*=3), ^*^P=0.028; Bonferroni-corrected *post hoc* test, data are expressed as mean±s.d. (upper); RT-PCR analysis of CA-IX, VEGF, HO-I, KDR, c-MET, HIF-1*α* and *β*2 *μ*m RNA level (middle); Western blot analysis of CA-IX, VEGF, HIF-1*α*, p53 and *β*-actin (lower). (**C**) pBabe-p53D stably transduced MCF-7 (MCF-7/p53D), exposed to <0.1% O_2_ for 24 h, and transiently transfected with p53Arg, p53Pro or (-) pCMS-GFP vector: cell death analysis (*n*=3), ^*^P=0.035; Bonferroni-corrected *post hoc* test, data are expressed as mean±s.d. (upper); RT-PCR analysis of CA-IX, VEGF, HO-I and *β*2 *μ*m RNA level (lower). Transfection efficiency was evaluated by fluorescent microscopy analysis of GFP positive cells (data not shown).

On the basis of the above results, it was reasoned that the p53Arg allele may be preferentially retained in heterozygote tumour cells owing to a survival advantage in presence of hypoxia. Following this hypothesis, we analysed the p53 codon72 genotype of 20 clones, obtained by the long-term exposure of the p53Arg/Pro heterozygote MCF-7 cell line to cytotoxic concentrations of DFX (see Supplementary Figure 1). PCR analysis revealed that all of the 20 clones obtained carried the p53Arg, but not the p53Pro allele (Supplementary Figure 1). HYPO-7 cells, the fastest growing clone, was further tested for the loss of heterozigosity at the p53 locus, by assessing the Intron I pentanucleotide repeat. The analysis confirmed that only one of the two allele present in parental MCF-7 cells was detectable in HYPO-7 cells (Figure 3A, upper panel). Interestingly, HYPO-7 cells maintained higher expression level of CA-IX, VEGF, HO-I, KDR mRNA than parental MCF-7 cells, even after several months of culture in absence of DFX (Figure 3A, lower panel, and Supplementary Figure 2). We then tested the capacity of the p53 codon 72 alleles to modulate the response to hypoxia in HYPO-7 cells. We found that, in the presence of <0.1% O_2,_ the p53Pro allele transiently transfected cells exhibited a higher rate of cell death, (Figure 3B, upper panel), lower levels of CA-IX, VEGF, HO-I and KDR mRNA (Figure 3B, middle panel), as well as a lower level of HIF-1*α* protein (Figure 3B, lower panel), compared to the p53Arg allele-transfected ones. To test further whether the p53 codon 72 alleles differ with regard to hypoxia survival in a MCF-7 genetic background, we transiently transfected pBabePuro-p53D stably-transduced MCF-7 cells with p53Pro/p53Arg alleles. In line with the results above, in the presence of <0.1% O_2_ the p53Pro allele elicited a higher degree of cell death, as well as lower levels of CA-IX, VEGF, HO-I mRNA, compared to the p53Arg allele (Figure 3C). These data suggest that the loss of the p53Pro allele in p53 codon 72 heterozygous cancer cells is associated with a survival advantage in presence of hypoxia.

## DISCUSSION

The present study provides evidence that the p53Pro allele of codon 72 locus confers a survival disadvantage to cancer cells in presence of hypoxia. This enhanced cell death inducing activity of the p53Pro allele in the presence of hypoxia, correlates with the lack of upregulation of a variety of genes (e.g., CA-IX, VEGF, c-MET, HO-I, KDR), whose promoters contain the consensus sequence for the hypoxia-induced transcription factor HIF-1*α* (Lee *et al*, 1997; Pal *et al*, 2001; Pennacchietti *et al*, 2003; Kaluzova *et al*, 2004). Accordingly, we found that HIF-1*α* protein and/or mRNA are decreased in cells transfected with the p53Pro allele. In this regard, it has been found that p53 downregulates HIF-1*α* at the protein level, but also that p53 −/− cells exposed to hypoxia show an increase in HIF-1*α* mRNA level in respect to p53 wild-type cells (Alarcon *et al*, 1999; Ravi *et al*, 2000; Nieminen *et al*, 2005). Hence, the available data support the hypothesis of a multilevel regulation of HIF-1*α* gene expression by p53. In this paper, we also show that the exposure of arginine/proline heterozygote MCF-7 cells to the hypoxia-mimetic drug Desferoxamine, yields the outgrowth of arginine hemi-zygote clones. We propose that these results support the notion that the p53Pro allele confers a growth disadvantage in a hypoxic environment. Accordingly, the reintroduction of the p53Pro, but not the p53Arg allele in hypoxia selected cells downregulates hypoxia response genes and promotes hypoxia-induced cell death. At present, it is not clear whether the p53Arg carrier, hypoxia selected clones are a sub-population that is already present among parental MCF-7 cells, or arise as a consequence of a hypoxia-induced genomic DNA damage. It is worth noting, however, that no p53Pro hemi-zygote cells were obtained from MCF-7 cells exposed to DFX (data not shown). In conclusion, we propose that the result here presented may provide a functional explanation for the preferential retention of the p53Arg allele (the preferential loss of the p53Pro) in tumour tissues of p53 codon 72 heterozygote individuals. Moreover, these data may provide a functional cue for explaining the recently reported reduction *in vivo* of the amount of spontaneous cell death in the p53Arg-retaining tumours arisen in heterozygote individuals (Schneider-Stock *et al*, 2004). Finally, we suggest that our results contribute to explain the controversial association between p53 codon 72 polymorphism in cancer. Indeed, the p53Arg allele, though provides survival advantage to cancer cells in the presence of hypoxia, induces a higher susceptibility to cell death in absence of such a stress condition (this paper, Bonafe *et al*, 2002; Dumont *et al*, 2003; Bonafe *et al*, 2004). This behaviour adds up to a picture of the same genotype having different effects in tumour malignant progression depending on different microenvironmental conditions, for example, the oxygenation level.
