# Supplementary material for: The p53 codon 72 proline allele is endowed with enhanced cell-death inducing potential in cancer cells exposed to hypoxia
Source: Br J Cancer. 2007 Apr 3;96(8):1302–8. doi: 10.1038/sj.bjc.6603723 (PMC2360160; doi:10.1038/sj.bjc.6603723)
Supplement: Supplementary Figure 1 [file 6603723x1.ppt]

## Slide 1
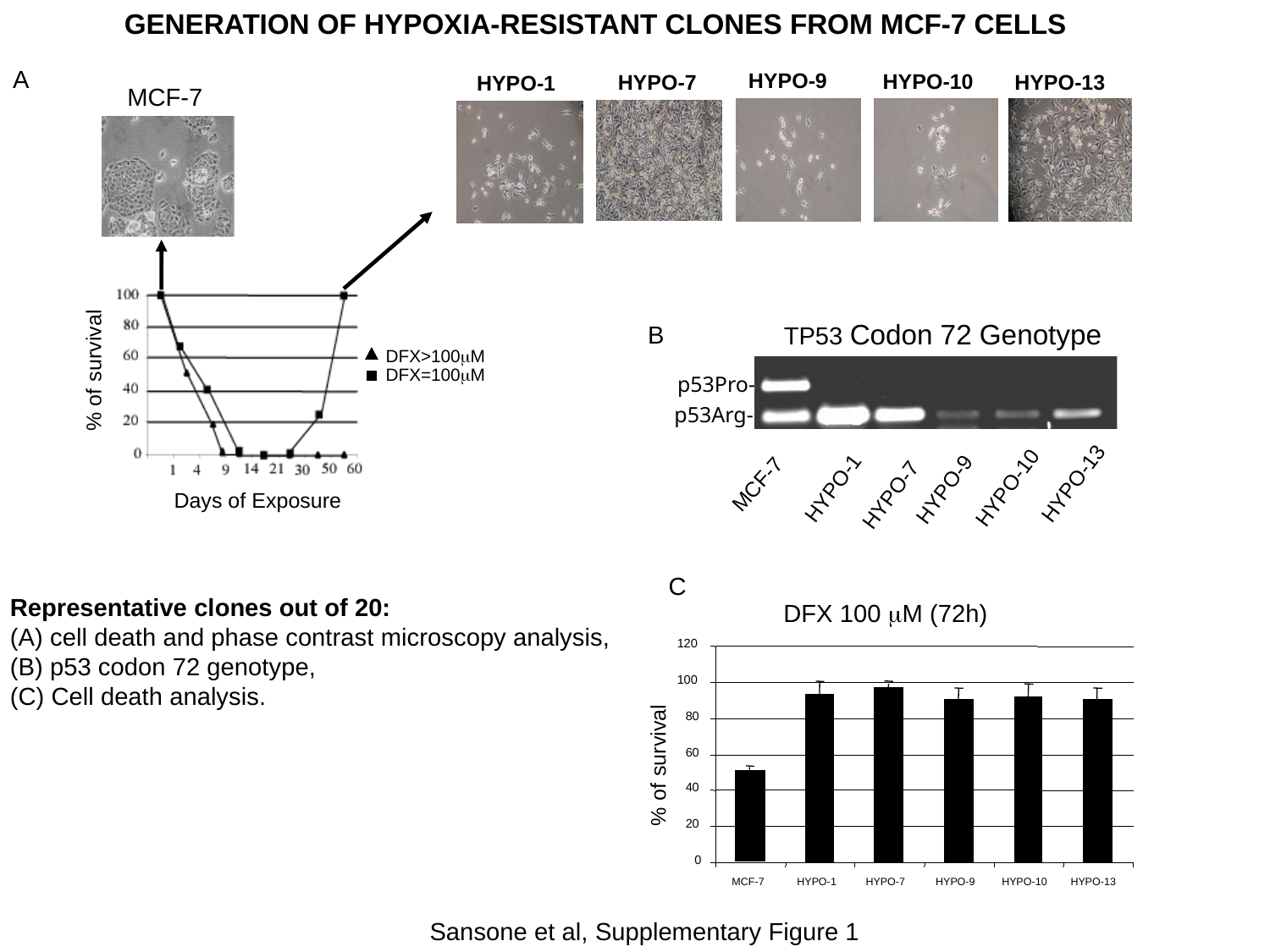

GENERATION OF HYPOXIA-RESISTANT CLONES FROM MCF-7 CELLS
A
HYPO-9
HYPO-10
HYPO-7
HYPO-13
HYPO-1
MCF-7
 TP53 Codon 72 Genotype
B
DFX>100μM
% of survival
DFX=100μM
p53Pro-
p53Arg-
HYPO-13
MCF-7
HYPO-10
HYPO-1
HYPO-9
HYPO-7
Days of Exposure
C
Representative clones out of 20:
(A) cell death and phase contrast microscopy analysis,
(B) p53 codon 72 genotype,
(C) Cell death analysis.
DFX 100 μM (72h)
120
100
80
% of survival
60
40
20
0
MCF-7
HYPO-1
HYPO-7
HYPO-9
HYPO-10
HYPO-13
Sansone et al, Supplementary Figure 1
