# Supplementary material for: The p53 codon 72 proline allele is endowed with enhanced cell-death inducing potential in cancer cells exposed to hypoxia
Source: Br J Cancer. 2007 Apr 3;96(8):1302–8. doi: 10.1038/sj.bjc.6603723 (PMC2360160; doi:10.1038/sj.bjc.6603723)
Supplement: Supplementary Figure 2 [file 6603723x2.ppt]

## Slide 1
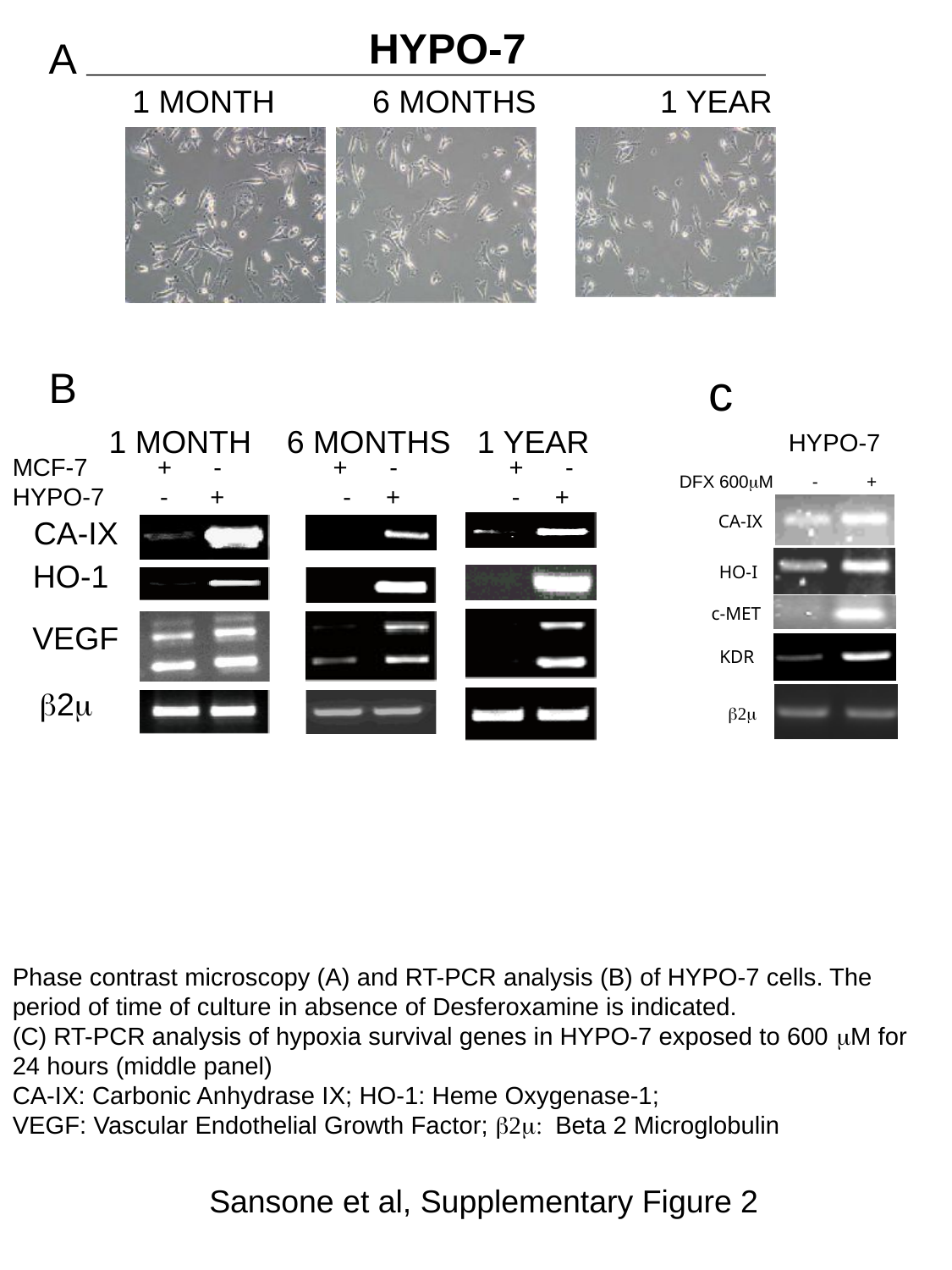

HYPO-7
A
1 MONTH 6 MONTHS 1 YEAR
B
c
1 MONTH 6 MONTHS 1 YEAR
HYPO-7
MCF-7 + - + - + -
HYPO-7 - + - + - +
DFX 600μM - +
CA-IX
CA-IX
HO-1
HO-I
c-MET
VEGF
KDR
β2

Phase contrast microscopy (A) and RT-PCR analysis (B) of HYPO-7 cells. The period of time of culture in absence of Desferoxamine is indicated.
(C) RT-PCR analysis of hypoxia survival genes in HYPO-7 exposed to 600 μM for
24 hours (middle panel)
CA-IX: Carbonic Anhydrase IX; HO-1: Heme Oxygenase-1;
VEGF: Vascular Endothelial Growth Factor;  Beta 2 Microglobulin
Sansone et al, Supplementary Figure 2
